# Multidrug‐resistant *Stenotrophomonas maltophilia* in residential aged care facilities: An emerging threat

**DOI:** 10.1002/mbo3.1409

**Published:** 2024-04-29

**Authors:** Sylvia A. Sapula, Bradley J. Hart, Naomi L. Siderius, Anteneh Amsalu, Jack M. Blaikie, Henrietta Venter

**Affiliations:** ^1^ Health and Biomedical Innovation, UniSA Clinical and Health Sciences University of South Australia Adelaide South Australia Australia; ^2^ Department of Medical Microbiology University of Gondar Gondar Ethiopia

**Keywords:** aged care, antimicrobial resistance, biocide tolerance, multidrug resistance, older persons, selective pressure

## Abstract

*Stenotrophomonas maltophilia* is a multidrug‐resistant (MDR), Gram‐negative bacterium intrinsically resistant to beta‐lactams, including last‐resort carbapenems. As an opportunistic pathogen, it can cause serious healthcare‐related infections. This study assesses the prevalence, resistance profiles, and genetic diversity of *S. maltophilia* isolated from residential aged care facilities (RACFs). RACFs are known for their overuse and often inappropriate use of antibiotics, creating a strong selective environment that favors the development of bacterial resistance. The study was conducted on 73 *S. maltophilia* isolates recovered from wastewater and facility swab samples obtained from three RACFs and a retirement village. Phenotypic and genotypic assessments of the isolates revealed high carbapenem resistance, exemplifying their intrinsic beta‐lactam resistance. Alarmingly, 49.3% (36/73) of the isolates were non‐wild type for colistin, with minimum inhibitory concentration values of > 4 mg/L, and 11.0% (8/73) were resistant to trimethoprim‐sulfamethoxazole. No resistance mechanisms were detected for either antimicrobial. Genotypic assessment of known lineages revealed isolates clustering with Sm17 and Sm18, lineages not previously reported in Australia, suggesting the potential ongoing spread of MDR *S. maltophilia*. Lastly, although only a few isolates were biocide tolerant (2.7%, 2/73), their ability to grow in high concentrations (64 mg/L) of triclosan is concerning, as it may be selecting for their survival and continued dissemination.

## Graphical abstract

This study marks the first assessment of the prevalence, resistome, and genomic characteristics of multidrug‐resistant (MDR) *Stenotrophomonas maltophilia* in residential aged care facilities. MDR *S. maltophilia* were found in all sampled facilities, with colistin non‐wild type and trimethoprim‐sulfamethoxazole resistant isolates recovered. Many isolates also exhibited high tolerance to triclosan and benzalkonium chloride. Notably, our findings indicate the continued emergence of *S. maltophilia*, with lineages not previously identified in Australia observed in this study. This underscores the urgent need for comprehensive surveillance and effective control measures.

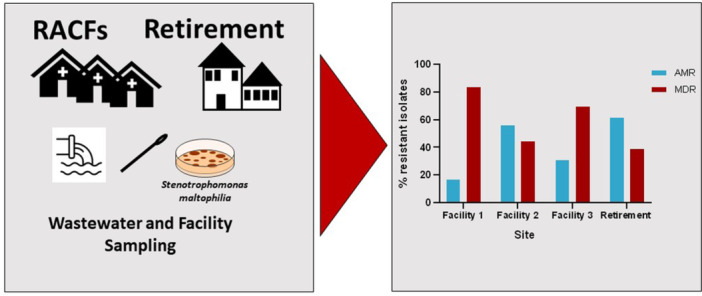

## 1 INTRODUCTION

*Stenotrophomonas maltophilia* is a Gram‐negative ubiquitous bacterium found in water, soil, plants, and food as well as healthcare settings such as hospitals (Brooke, 2012). Its main habitat is postulated to be soil, with the plant rhizosphere identified as its natural environment where it outcompetes other rhizospheric bacteria (Alavi et al., 2014; Youenou et al., 2015). In healthcare settings, the frequency of isolation continues to increase with *S. maltophilia* found to be predominantly associated with respiratory infections, such as those inherent to cystic fibrosis (Looney et al., 2009; Vidigal et al., 2014), and bloodstream infections (Chang et al., 2014; Garazi et al., 2012). *S. maltophilia* infections are also associated with high mortality rates ranging from 21% to 69% (Ahlström et al., 2022; Paez & Costa, 2008). Risk factors associated with *S. maltophilia* infections include an immunocompromised host, co‐morbidities, chronic respiratory disease, presence of an indwelling catheter and prior use of antibiotics (Brooke, 2012; Goss et al., 2002). *S. maltophilia* is also commonly detected in polymicrobial infections with other bacteria including *Pseudomonas aeruginosa*, *Klebsiella pneumoniae* and *Acinetobacter baumannii*, complicating treatment options (McDaniel et al., 2022; Wang et al., 2022). Co‐infections with *P. aeruginosa* are linked with higher mortality rates and may stem from the ability of both organisms to produce biofilms in the respiratory tract, making treatment notoriously difficult (Yin et al., 2017). The use of extended‐spectrum antibiotics to treat polymicrobial infections may also result in the selection of multidrug‐resistant (MDR) *S. maltophilia* strains (Adegoke et al., 2017).

*S. maltophilia* is considered to be an emerging opportunistic pathogen which is increasing in frequency of isolation in healthcare environments (Brooke, 2021). However, community‐acquired infections have also been reported and are on the rise (Chang et al., 2014). The success of *S. maltophilia* as an opportunistic pathogen stems from its intrinsic resistance to commonly used antibiotics, including carbapenems and aminoglycosides (Xun et al., 2014), but acquired resistance against fluoroquinolones and/or colistin is also known to occur (García‐León et al., 2014). As such, *S. maltophilia* is considered an MDR organism (Crossman et al., 2008; Ryan et al., 2009). Due to its MDR status, treatment options for *S. maltophilia* infections are limited, with trimethoprim‐sulfamethoxazole, also known as co‐trimoxazole, considered the treatment of choice, followed by the use of quinolones and ticarcillin/clavulanate (Gil‐Gil et al., 2020). However, resistance against trimethoprim‐sulfamethoxazole continues to develop worldwide, with isolates found carrying the class 1 integron and the insertion element common region (ISCR) linked *sul* genes, which confer high‐level trimethoprim‐sulfamethoxazole resistance (Chung et al., 2015; Elsheredy et al., 2022; Toleman et al., 2007). As a result of this emerging resistance, and as the use of trimethoprim‐sulfamethoxazole can be associated with treatment‐limiting toxicity, the fluoroquinolone antibiotic, levofloxacin, has been used as an alternative treatment option (Sarzynski et al., 2022). Genetic mutations also serve as a major cause of acquired resistance in *S. maltophilia* (Gil‐Gil et al., 2020 *)*. Examples include mutations in the regulators of the multidrug efflux pumps SmeDEF, SmeVWX and SmeGH. When overexpressed, the former two are associated with trimethoprim‐sulfamethoxazole and tigecycline resistance (García‐León et al., 2014; Sánchez & Martínez, 2018) and the latter, with fluoroquinolone resistance (Li, Zhang, et al., 2019).

Genome‐based assessment of *S. maltophilia* revealed continued evolution and adaption to new environments such as clinical settings (Gröschel et al., 2020; Ryan et al., 2009). These studies have also shown that the distinction between environmental and clinical strains is not well defined as both clinical and environmental isolates are intrinsically resistant, virulent and able to adapt, colonize and cause infections in humans.

*S. maltophilia* represents a growing global public health concern due to their MDR phenotype, ability to adapt, presence in co‐infections, increasing prevalence in healthcare settings and limited treatment options. Although numerous studies have been carried out assessing *S. maltophilia* isolated from hospital settings (Flores‐Treviño et al., 2014; Mendes et al., 2020; Vidigal et al., 2014), none have analyzed isolates from residential aged care facilities (RACFs). However, the elderly (>73 years of age) is a high‐risk group for the acquisition of *S. maltophilia* infections (Hu et al., 2018; Liang et al., 2014; Yue et al., 2021). This risk is exasperated by the high and often inappropriate antibiotic use in RACFs (Lim et al., 2015; Stuart et al., 2012). Like many other healthcare environments, RACFs are also heavy users of biocides, however, recent studies have shown that the use of biocides may lead to cross‐resistance, with multidrug efflux pumps playing a key role in this development (Amsalu et al., 2020). This was exemplified in *S. maltophilia*, with triclosan selection for *S. maltophilia* mutants overexpressing the SmeDEF efflux pump (Hernández et al., 2011; Sánchez et al., 2015). Since prior exposure to antibiotics and possibly biocides select for MDR *S. maltophilia*, RACFs may be exemplary environments for the selection and potential transmission of this organism.

This study aimed to assess the phenotypic and genotypic resistance of *S. maltophilia* isolated from RACFs and Retirement living. This is the first study to evaluate the resistome of *S. maltophilia* isolated from RACFs and highlight the capacity of these organisms to adapt to a changing and selective environment. In addition, as biocides are commonly used in these settings, tolerance to a few commonly used biocides was also evaluated. Finally, a comparison of *S. maltophilia* isolated from RACFs with environmental strains was also conducted and their monophyletic lineages (Gröschel et al., 2020) were determined.

## 2 METHODS

### 2.1 Sampling

*S. maltophilia* isolates included in this study originated from three aged care facilities (Facility 1, 170 beds; Facility 2, 70 beds; Facility 3, 58 beds) and one independent living facility (Retirement, 38 beds) in Adelaide, Australia. Isolates were obtained from facility swabs and wastewater samples collected over 18 months between October 2019 and February 2021. Sterile swabs, used to swab facility surfaces such as toilets, sinks, and door handles as well as common areas such kitchens and cafes, were transported to the laboratory on ice, re‐suspended in 200 µL of peptone water supplemented with 20% (*v*/*v*) glycerol and stored at −80°C.

Wastewater was sampled from two aged care facilities (Facility 1 and 2) and one Retirement village in Adelaide. Sampling of Facility 3 wastewater could not be carried out due to a lack of access to a sampling point. Wastewater samples were collected at five different time points at approximately 3‐month intervals, with a total of 15 samples collected over this time frame. Grab samples (approximately 200 mL) were collected every hour over a 10 h period from a collection point which captured all the wastewater from each RACF. The samples were then pooled for analysis. Sampled wastewater was transported to the laboratory on ice, stored at 4°C and processed on the day of collection. Environmental samples were collected from the river Torrens, a local river in Adelaide, where three 100 mL samples were collected and transported on ice to the laboratory for processing. All river samples were processed on the day of collection.

### 2.2 Isolation of *S. maltophilia*

Antimicrobial‐resistant *S. maltophilia* isolates were screened on selective and differential media. Selection was carried out in the presence of meropenem as *S. maltophilia* is intrinsically resistant to carbapenems. Swabs, wastewater and river samples (100 µL) were plated onto Tergitol‐7 media (CM0793, Oxoid; Thermo Fisher Scientific) supplemented with meropenem at 2 mg/L and onto CHROMagar™ MDR Acinetobacter plates. Cultures were incubated at 25°C for 48 h. Following colony purification, identification was carried out using matrix‐assisted laser desorption/ionization time‐of‐flight mass spectrometry (MALDI‐TOF) (Bruker Daltonik GmbH). Confirmed *S. maltophilia* isolates were stored at −80°C in Tryptone Soya broth (CM0129, Oxoid; Thermo Fisher Scientific) supplemented with 20% (*v*/*v*) glycerol.

### 2.3 Antimicrobial susceptibility testing

Antimicrobials for which susceptibility was assessed in this study included cefepime, ceftazidime, gentamicin, minocycline, meropenem, imipenem, trimethoprim‐sulfamethoxazole, colistin, triclosan, chlorhexidine (ChemSupply) benzalkonium chloride and ciprofloxacin (Sigma‐Aldrich).

Minimum inhibitory concentrations (MIC) to antimicrobials were assessed using the microbroth dilution method (ISO 20776‐1) as recommended by the European Committee on Antimicrobial Susceptibility Testing (EUCAST) guidelines (EUCAST, 2003). *Pseudomonas aeruginosa* ATCC 27853 was used as a quality control strain in each experiment.

Where possible epidemiological cut‐off (ECOFF) values, as reported by the European Committee on Antimicrobial Susceptibility Testing (https://mic.eucast.org/search/: accessed 2nd February 2023) were used to distinguish wild‐type from non‐wild‐type isolates. The use of “wild type” and “non‐wild type” are used here to reflect different populations within the study (based on the ECOFF values) and to highlight that these are not used as predictors of clinical efficacy. In addition, available Clinical and Laboratory Standard Institute (CLSI) breakpoints for *S. maltophilia* (Clinical and Laboratory Standard Institute [CLSI], 2021 *)*, were used to identify resistant isolates.

As ECOFF values have not been determined for biocides, these were also assessed in this study, following the methodology described above. ECOFF (with 95% cut‐off) values were then calculated using the ECOFF find XL 2010 program (https://clsi.org/meetings/microbiology/ecoffinder/ accessed 2nd February 2023).

### 2.4 Genomic DNA isolation

Genomic DNA was extracted using the MN NucleoSpin®Microbial DNA kit (Machery‐Nagel GmbH and Co. KG), following the manufacturer's instructions. DNA quantity and quality were assessed using a Cytation5 imaging reader (BioTek Instruments). Extracted genomic DNA was stored at −20°C.

### 2.5 Whole genome sequencing (WGS) and data analysis

WGS was performed on a subset of *S. maltophilia* isolates selected to include those with high‐level resistance, with a sensitive isolate included to serve as a control. Sequencing was carried out at SA Pathology using the Illumia NextSeq platform. Sequencing libraries were prepared using the Nextera XT DNA library preparation kit (Illumina Inc.) as per the manufacturer's instructions. WGS was performed on the Illumina NextSeq. 550 platform with the NextSq 500/550 Mid‐Output kit v2.5 (300 cycles) (Illumina Inc.). Raw 150 bp paired‐end reads were utilized as input data for the TORMES v.1.3.0 (Quijada et al., 2019) pipeline for the analysis of whole bacterial genomes. As part of this process, filtering of reads was performed via Prinseq v.0.20.4 (Schmieder & Edwards, 2011). Assembly of cleaned reads was carried out with SPAdes v.3.15.2 (Bankevich et al., 2012). Taxonomic identification and typing of sequences was carried out by Kraken2 v.2.0.9 (Wood et al., 2019) and MLST v.2.19.0 (T. Seemann, https://github.com/tseemann/mlst), respectively, which uses the PubMLST database (Jolley & Maiden, 2010).

Antimicrobial resistance genes were screened using ABRicate v.1.0.1 (T. Seemann, https://github.com/tseemann/abricate) utilizing databases such as ResFinder (Zankari et al., 2012), the Comprehensive Antibiotic Resistance Database (CARD) (McArthur et al., 2013) and the ARG‐ANNOT (Gupta et al., 2014), database. Additional software such as PointFinder v.3.1.0 (Zankari et al., 2017) was used to screen for chromosomal point mutations, and PlasmidFinder v.2.1.6 (Carattoli et al., 2014) was used for plasmid replicon screening.

Annotation and gene prediction were performed using PROKKA v.1.14.6 (Seemann, 2014). Pangenome analysis and generation of approximately‐maximum‐likelihood phylogenetic trees was carried out using Roary v.3.13.0 (Page et al., 2015) with gene core allocation requiring a presence in 99% or greater of all sequences analyzed. Associated phylogenetic trees were generated using FastTree v.2.1.10 (Price et al., 2010). Further analysis including the determination of average nucleotide identity (ANI) percentages and subsequent figure generation was completed using Anvio v.7.1 (Eren et al., 2021). Finally, the Interactive Tree of Life (ITOL) v.4 (Letunic & Bork, 2019), was utilized in the annotation of the phylogenetic tree of sequenced isolates.

### 2.6 Statistical analysis

Principal component analysis (PCA) was performed in R studio v.1.2.5033 and used here to visually compare the distribution of *S. maltophilia* isolates recovered from different sites (facilities). Bar graphs were generated using GraphPad Prism v9.

## 3 RESULTS

### 3.1 *S. maltophilia* isolated from RACFs display resistance against trimethoprim‐sulfamethoxazole and high MICs against colisitin and gentamycin

Environmental samples, such as wastewater and facility surface swabs, were used to evaluate the presence and resistance patterns of *S. maltophilia* isolated from three RACFs and one Retirement village. It is difficult to assess the levels of resistance to beta‐lactams as no ECOFF or clinical breakpoints exist for compounds of this class, however, 87.7% (*n* = 64/73) of isolates assessed in this study displayed high MICs of ≥ 256 mg/L against imipenem and 41.1% (n = 30/73) against meropenem (Table 1). Although *S. maltophilia* are considered intrinsically carbapenem‐resistant, they are not considered to be intrinsically resistant to ceftazidime and cefepime (Mojica et al., 2022). EUCAST does not provide an MIC breakpoint or an ECOFF for cefepime for *S. maltophilia*, however, it provides an ECOFF of > 8 mg/L for ceftazidime. Ceftazidime is also one of only seven antibiotics for which CLSI breakpoints are provided, with ≥ 32 mg/L reported to indicate resistance (CLSI, 2021). Using these values, 72.6% (*n* = 53/73) of the isolates assessed in this study were found to be ceftazidime non‐wild type and 52.1% (*n* = 38/73) resistant. Finally, 35.6% (*n* = 26/73) and 26.0% (*n* = 19/73) of isolates displayed high MICs of ≥ 128 mg/L against cefepime and ceftazidime, respectively.

**Table 1:** MIC distribution for 73 *Stenotrophomonas. maltophilia* isolates recovered from facility swabs and wastewater sampled from three RACFs and a Retirement village.

	Number of *S. maltophilia* Isolates with MICs (mg/L) at:														
	0.03	0.06	0.125	0.25	0.5	1	2	4	8	16	32	64	128	256	>256
MER	0	0	0	0	0	0	0	0	2	0	6	11	24	28	2
IMI	0	0	0	0	0	0	0	0	0	0	1	2	6	25	39
FEP	0	0	0	1	0	1	1	3	4	5	12	20	24	2	0
CAZ	0	0	0	0	3	7	5	5	10	5	11	8	17	2	0
CIP	0	0	2	8	22	18	14	7	2	0	0	0	0	0	0
MIN	1	6	16	24	11	11	3	0	1	0	0	0	0	0	0
COL	0	0	0	0	8	5	12	12	8	17	4	3	4	0	0
GEN	0	0	0	0	7	14	11	10	12	7	6	3	3	0	0
SXT	3	3	12	11	9	13	13	7	1	0	0	0	0	0	0

Abbreviations: CAZ, ceftazidime; CIP, ciprofloxacin; COL, colistin; FEP, cefepime; GEN, gentamicin; IMI, imipenem; MER, meropenem; MIN, minocycline; SXT, trimethoprim‐sulfamethoxazole.

*Note*: Vertical black lines indicate EUCAST ECOFF values. Green shading—MICs higher than the ECOFF values listed for *S. maltophilia*.

A lack of ECOFF or a clinical breakpoint also pertains to colistin, resulting in difficulties in establishing susceptibility and resistance status. As a result, studies assessing colistin in *S. maltophilia* use ECOFFs established for *Pseudomonas* sp. (ECOFF > 4 mg/L) in colistin susceptibility classification of *S. maltophilia* (Betts et al., 2014; EUCAST, 2022; Rodríguez et al., 2014). In this study, colistin MIC levels of > 4 mg/L were determined for 49.3% (*n* = 36/73) of isolates. Non‐wild type phenotypes were also observed for gentamicin, classified here as > 8 mg/L (based on *P. aeruginosa* ECOFF of > 8 mg/L), with 26.0% (*n* = 19/73) isolates observed to have MICs above this ECOFF value. Finally, 11.0% (*n* = 8/73) of isolates assessed in this study were be found to trimethoprim‐sulfamethoxazole non‐wild type, with an ECOFF value of > 2 mg/L. These isolates were also determined to be resistant as the CLSI MIC breakpoint for trimethoprim‐sulfamethoxazole is ≥ 4 mg/L. All isolates were susceptible to ciprofloxacin (ECOFF value > 16 mg/L) and only four isolates were found to be minocycline non‐wild type (ECOFF value > 1 mg/L). However, according to the CLSI breakpoint available for minocycline, with ≥ 16 mg/L determined for resistant *S. maltophilia*, these four isolates were sensitive, with their MICs falling below the established MIC breakpoint for this compound.

**Figure 1:**
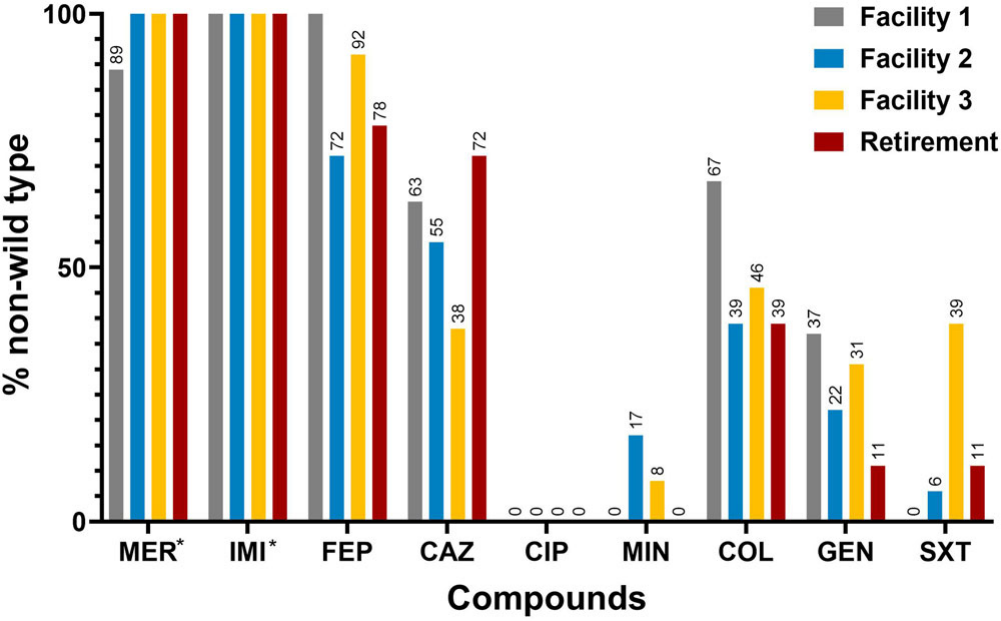
Percentage of total (*n* = 73) non‐wild type *S. maltophilia* isolates per facility and retirement village (Facility 1; *n* = 24, Facility 2; *n* = 18; Facility 3; *n* = 13, Retirement; *n* = 18). A non‐wild type phenotype for each isolate was determined using the following ECOFF values; ciprofloxacin > 16, ceftazidime > 8, minocycline > 1 and trimethoprim‐sulfamethoxazole > 4, as determined for *S. maltophilia* and cefepime > 8, colistin > 4 and gentamycin > 8, as determined for *Pseudomonas aeruginosa*. *With no ECOFF values available for imipenem and meropenem and owing to their intrinsic resistance against these compounds isolates with minimum inhibitory concentrations values above 32 mg/L were considered non‐wild type.

Assessment of the prevalence of isolates per facility revealed Facility 1 to harbor a high number of cefepime, ceftazidime, colistin and gentamicin non‐wild type isolates while Facility 3 represented the site from which a greater number of trimethoprim‐sulfamethoxazole non‐wild type *S. maltophilia* were isolated (Figure 1). Notably, 66.7% (*n* = 16/24) of Facility 1 isolates showed high (MIC ≥ 128 mg/L) levels of cefepime resistance, and 16.7% (*n* = 4/24) displayed high colistin resistance (MIC ≥ 128 mg/L). Such high colistin resistance was not observed for any other facility. Finally, trimethoprim‐sulfamethoxazole resistance (MIC ≥ 4 mg/L) was predominantly observed in isolates (38.5%, *n* = 5/13) recovered from Facility 3.

**Figure 2:**
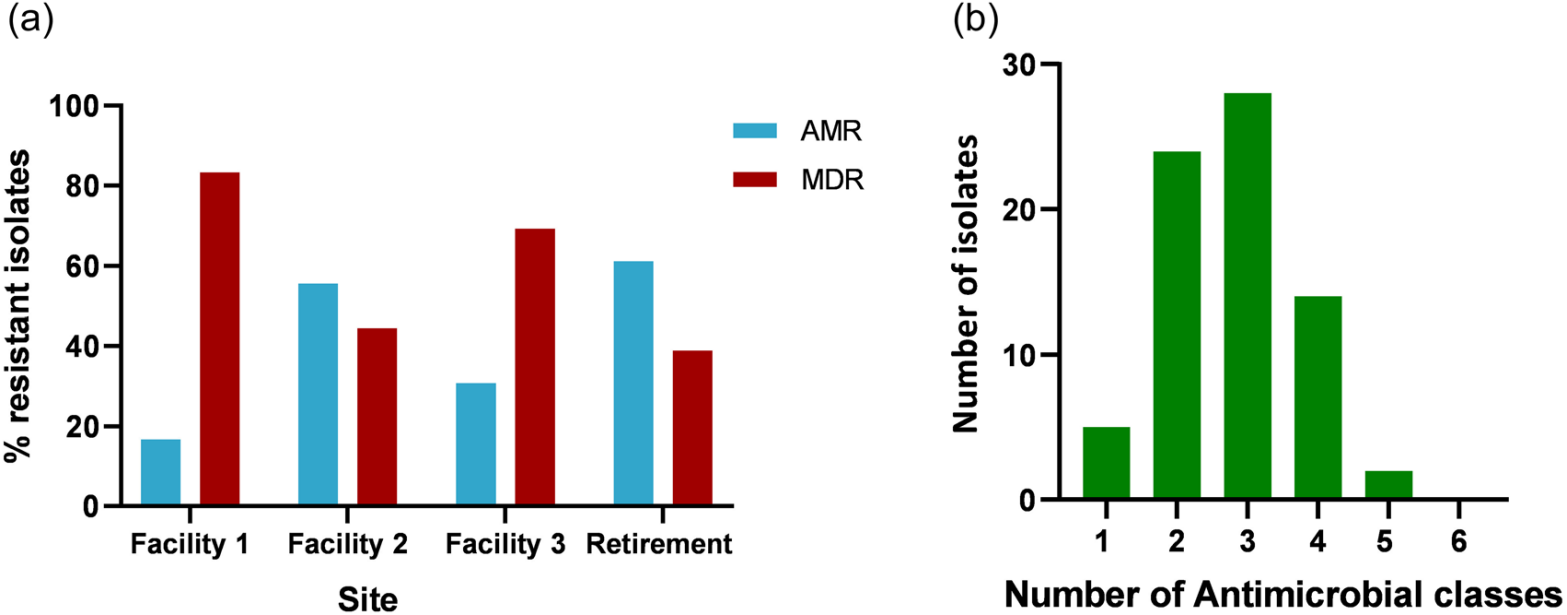
(a) Percentage of multidrug‐resistant (MDR) and non‐MDR *Stenotrophomonas maltophilia* isolates per residential aged care facility and Retirement. (b) Number of antibiotics (total *n* = 6) each *S. maltophilia* isolate was resistant against.

**Figure A1:**
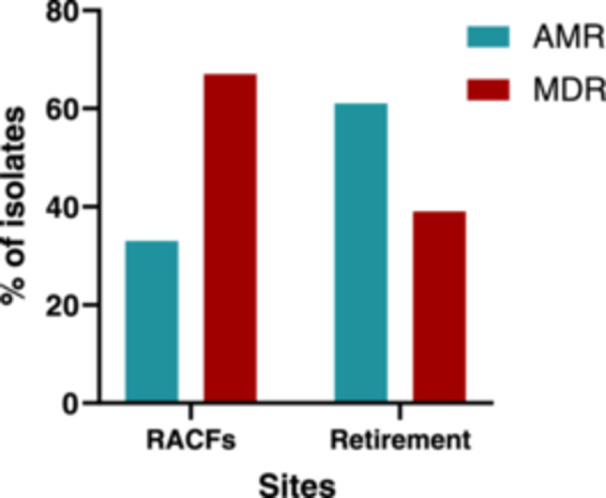
Percentage of multidrug‐resistant (MDR) and non‐MDR *Stenotrophomonas maltophilia* isolates isolated from three residential aged care facilities and a Retirement village.

Of the 73 *S. maltophilia* isolates assessed in this study, 60.3% (*n* = 44/73) exhibited an MDR phenotype (resistant or showing a non‐wild type phenotype to antibiotics in 3 or more classes) (Figure 2a,b). Of these, Facility 1 was found to carry the largest number (83.3%, *n* = 20/24) of MDR *S. maltophilia* isolates. In contrast, Facility 2 was found to carry fewer MDR isolates (44.4%, *n* = 8/18) a trend also observed for Retirement samples, with 38.9% (*n* = 7/18) of isolates displaying a MDR phenotype. A comparison between RACFs and Retirement samples (Figure A1), revealed the proportion of isolates displaying a MDR profile was greater for RACFs (67.3%, *n* = 37/55) than for Retirement isolates (38.9%, *n* = 7/18).

**Figure 3:**
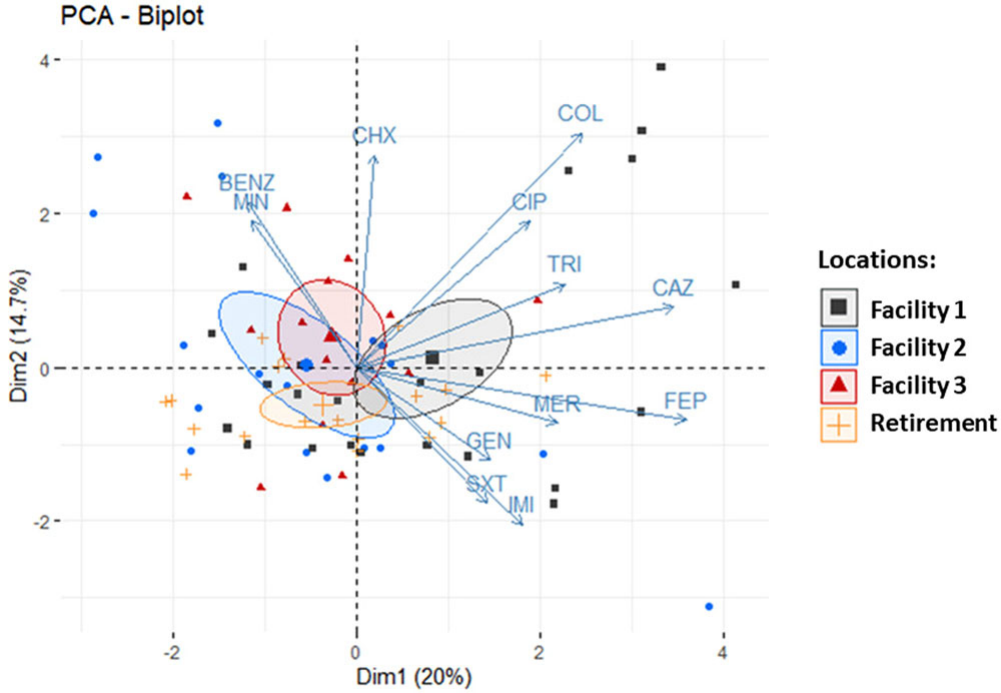
Principal component analysis (PCA) plot showing the clustering of resistant *Stenotrophomonas maltophilia* isolates recovered from Facility 1 (black; *n* = 24), Facility 2 (blue; *n* = 18), Facility 3 (red; *n* = 13) and a Retirement village (yellow; *n* = 18). Ellipses were drawn at a confidence level of 0.95.

PCA was used to assess the distribution of all isolates based on their resistance profile (Figure 3). The bi‐plot analysis reveals the clustering of isolates per site of isolation and co‐clustering of isolates shown to be resistant to a number of different antibiotics. Of these, Facility 1 isolates appear to make up a large part of this cluster, indicating that this facility harbors an environment which is more selective for MDR *S. maltophilia*.

### 3.2 Biocide susceptibility of *S. maltophilia* isolates

**Figure A2:**
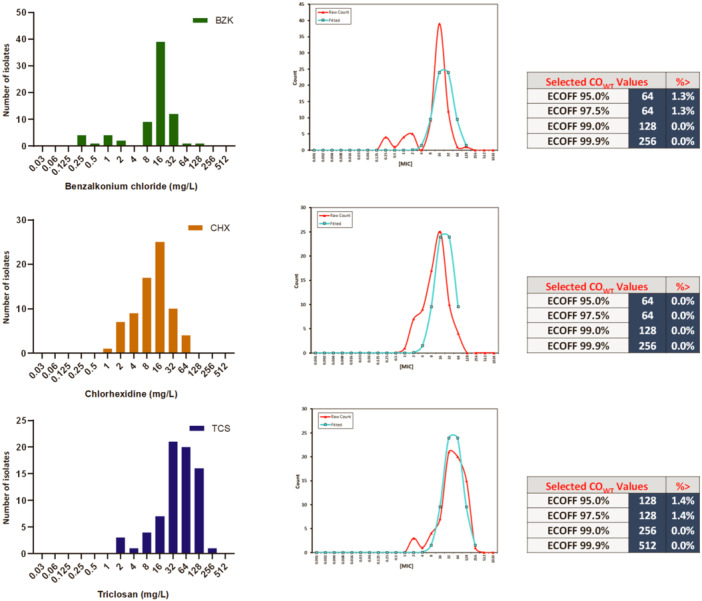
*Stenotrophomonas maltophilia* susceptibility patterns to benzalkonium chloride (BZK), chlorhexidine (CHX) and triclosan (TCS) allowed for the establishment of epidemiological cut‐off (ECOFF) values with 64, 64, and 128 mg/L, respectively, determined as the ECOFF values for these compounds.

Although biocides are widely used for decontamination and disinfection in healthcare and non‐healthcare environments, tolerance to these is not frequently addressed in studies assessing *S. maltophilia*. With no ECOFF values available for biocides, it is difficult to define and attribute biocide tolerance in *S. maltophilia*, which can be defined as the ability to grow at concentrations above the ECOFF. Following methodology used to assess biocide tolerance in clinically relevant microorganisms (Morrissey et al., 2014), MIC results obtained for benzalkonium chloride, chlorhexidine and triclosan (Table 2) were used to establish ECOFF values for these compounds (Figure A2) for *S. maltophilia*. According to these results, isolates analyzed in this study were sensitive to all of the biocides assessed, except one isolate recovered from Facility 2 which was shown to be benzalkonium chloride tolerant (ECOFF 64 mg/L) with a MIC of 128 mg/L, and one Facility 3 isolate shown to be triclosan tolerant (ECOFF 128 mg/L) with a MIC of 256 mg/L.

**Table 2:** Biocide MIC distribution for 73 *Stenotrophomonas maltophilia* isolates recovered from three residential aged care facilities and a Retirement village.

Number of *S. maltophilia* isolates with MICs (mg/L) at:															
	**0.03**	**0.06**	**0.125**	**0.25**	**0.5**	**1**	**2**	**4**	**8**	**16**	**32**	**64**	**128**	**256**	**512**
BZK	0	0	0	4	1	4	2	0	9	39	12	1	1	0	0
CHX	0	0	0	0	0	1	7	9	17	25	10	4	0	0	0
TCS	0	0	0	0	0	0	3	1	4	7	21	20	16	1	0

*Note*: Vertical black lines indicate ECOFF values as established in this study. ECOFF values determined according to Turnidge et al. (2006).

Abbreviations: BZK, benzalkonium chloride; CHX, chlorhexidine; TCS, triclosan.

### 3.3 Genomic analysis of a subset of *S. maltophilia* reveals novel sequence types and a conserved resistome

To evaluate the resistome of the *S. maltophilia* isolates assessed in this study, whole genome sequencing (WGS) was carried out on a representative sample of MDR isolates from each RACF and Retirement village. Also included was isolate Sm6012, which was one of two isolates with carbapenem MIC values below 256 mg/L (imipenem 64 mg/L and meropenem 8 mg/L). This isolate was also one of the four isolates found to be minocycline non‐wild type (MIC 2 mg/L) and as such was included for further in‐depth analysis. Isolates Sm3119 and Sm0581 (both recovered from Facility 1) were found to have high MICs against colistin (MICs of 64 and 128 mg/L, respectively), while isolate Sm2128 (Retirement living isolate), was chosen for further analysis due to its high gentamicin resistance (MIC of 128 mg/L).

An additional two *S. maltophilia* isolates recovered from a river environment were also sequenced to evaluate how healthcare‐isolated strains compared to environmental strains. The two river isolates, Sm0008_R and Sm0010_R, were both MDR and were found to have high MICs against all of the antibiotics analyzed in this study except ciprofloxacin, minocycline and trimethoprim‐sulfamethoxazole **(**Table 3), a trend observed for many of the RACFs and Retirement isolates.

**Table 3:** Antibiotic and biocide minimum inhibitory concentration (MIC) profiles of sequenced *Stenotrophomonas maltophilia* isolates and reference strains K279a and R551‐3.

		Site of isolation	MIC (mg/L)											
			MER	IMI	FEP	CAZ	CIP	MIN	COL	GEN	SXT	BZK	CHX	TCS
Control strain	*P. aeruginosa* ATCC 27853	–	**1**	**2**	**2**	**4**	**0.25**	**16**	**1**	**2**	**>8**	**64**	**8**	**512**
Reference	K279a^a^	Clinical	32	256	4	8	2	0.25	8	16	≤20	ND	ND	ND
	R551‐3^a^	Environment	≥16	≥16	16	4	0.5	≤1	0.5	≤1	≤20	ND	ND	ND
	ECOFF	–	**ND**	**ND**	**ND**	**8**	**16**	**1**	**ND**	**ND**	**2**	**64** b	**128** b	**256** b
Facility 1	Sm3147	Wastewater	256	512	128	64	2	0.25	4	16	2	8	8	64
	Sm3119	Wastewater	512	512	128	256	1	0.5	64	16	0.5	16	32	128
	Sm0041	Facility	512	512	128	256	2	0.0625	16	8	0.125	16	8	32
	Sm0581	Facility	128	512	128	128	2	0.25	128	4	0.5	32	32	64
Facility 2	Sm6012	Wastewater	8	64	16	0.5	1	2	0.5	1	1	64	16	64
Facility 3	Sm5341	Facility	256	512	64	32	4	0.5	4	8	4	16	32	32
	Sm2017	Facility	256	512	64	8	1	1	8	16	4	32	16	16
Retirement	Sm3212	Wastewater	128	512	64	16	2	0.25	16	64	2	8	8	128
	Sm2128	Wastewater	256	256	128	128	0.25	0.25	32	128	4	16	16	32
River	Sm0008_R	River	256	512	128	128	2	0.25	264	16	0.125	16	16	16
	Sm0010_R	River	256	512	64	64	1	0.125	32	64	0.5	16	8	16

*Note*: Bold values indicate that MIC concentrations assessed.

Abbreviations: ATM, Aztreonam; BZK, benzalkonium chloride; CAZ, ceftazidime; CHX, chlorhexidine; CIP, ciprofloxacin; COL, colistin; FEP, cefepime; GEN, gentamicin; IMI, imipenem; LEV, Levofloxacin; MER, meropenem; MIN, minocycline; ND, not determined; SXT, trimethoprim‐sulfamethoxazole; TCS, triclosan.

^a^ MIC values for K279a and R551‐3 as reported by Crossman et al. (2008), Youenou et al. (2015).

^b^ Biocide ECOFF values as determined in this study.

**Figure 4:**
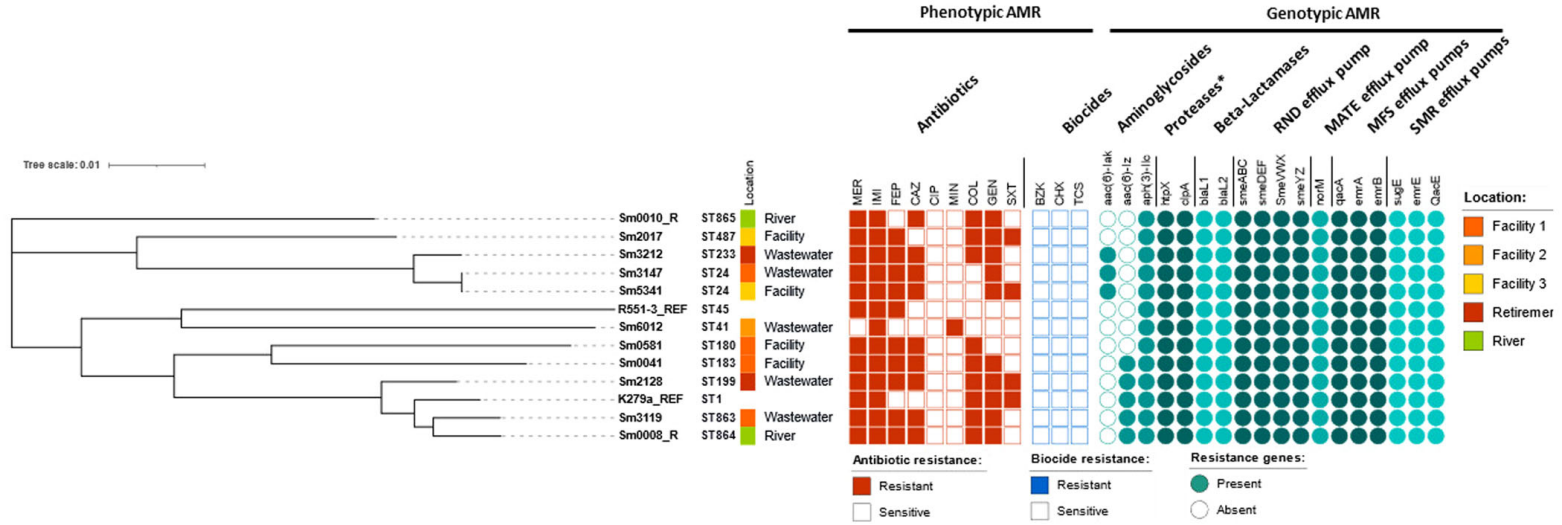
Phylogenetic tree from maximum‐likelihood analysis of the core genome alignments of 11 *Stenotrophomonas maltophilia* isolated from RACFs, Retirement and a river environment. The *S. maltophilia* K279a and R551‐3 isolates were included as reference strains representing a clinical (K279a) and an environmental (R551‐3) strain. Vertical columns demonstrate the (1) ST of each isolate, (2) location of isolation, (3) source, (4) AMR (red squares) and (5) biocide (blue squares) resistance profile (filled in representing a resistant phenotype). The resistance phenotype of each isolate is shown in teal circles (filled in representing the presence of the resistance determinant in that isolate). BZK, benzalkonium chloride; CAZ, ceftazidime; CHX, chlorhexidine; CIP, ciprofloxacin; COL, colistin; FEP, cefepime; GEN, gentamicin; IMI, imipenem; MER, meropenem; MIN, minocycline; SXT, trimethoprim‐sulfamethoxazole; TCS, triclosan. * Proteases implicated in aminoglycoside resistance (Mojica et al., 2022).

Core‐genome phylogenetic clustering carried on the sequenced and reference isolates K279a (clinical origin) (Crossman et al., 2008) and R551‐3 (environmental origin) (Lucas et al., 2008), revealed that the *S. maltophilia* isolate Sm3119, recovered from Facility 1 wastewater, and the environmental (river) isolate Sm0008_R clustered with K279a (Figure 4). Both Sm3119 and Sm0008_R are MDR isolates. The largely sensitive Sm6012 was the only isolate observed to cluster with the environmental R551‐3 reference strain. No apparent cluster trend was visible for the isolation sites; however, this may be due to the limited number of isolates sequenced and assessed in this study.

Resistome analysis of the sequenced isolates revealed resistance genes coding for aminoglycosidases (AAC(6′)‐lak, AAC(6′)‐lz and APH(3′)‐llc) and beta‐lactamases (L1 and L2 beta‐lactamases) in addition to efflux pumps belonging to the RND, MFS, MATE and SMR families (Figure 4). All of these contribute to high‐level intrinsic resistance which is characteristic of *S. maltophilia* (Brooke, 2014). In this study, of the 11 sequenced isolates, all were found to be carrying the *bla*_L1_ and *bla*_L2_ beta‐lactamase genes, however, Sm6012 displayed comparatively low levels of meropenem resistance. Meropenem is a substrate of the L1 enzyme, with L2 displaying negligible activity against this compound (Calvopiña et al., 2017). A multiple sequence alignment of the sequenced L1 enzymes (data not shown) revealed numerous uncharacterized amino acid variations within the L1 sequence of Sm6012, however, none within the active site. In addition to low meropenem resistance, Sm6012 was also cefepime and ceftazidime susceptible. An alignment of the L2 class A cephalosporinase (data not shown), also revealed sequence variations with this sequence, however, as determined for L1, no mutations were observed in the active site. As Sm6012 is susceptible to the cephalosporins tested in this study and displays comparatively lower levels of resistance against meropenem, susceptibility to these compounds may stem from lower expression levels of both L1 and L2, with studies showing common components controlling the expression of both enzymes (Avison, 2002). Interestingly, although susceptible to many of the compounds assessed in this study, this isolate displayed a non‐wild type phenotype for minocycline, with a MIC of 2 mg/L (ECOFF > 1 mg/L) observed for this compound. Minocycline susceptibility is common among *S. maltophilia* (Gibb & Wong, 2021), as was reflected in this study with 94.5% (*n* = 69/73) of isolates found to be minocycline susceptible. The non‐wild type phenotype observed for Sm6012 may be due to RND efflux pump overexpression, which has been associated with elevated minocycline resistance (Gould et al., 2013; Venter et al., 2015).

In addition to the L1 and L2 beta‐lactamases, *S. maltophilia* also harbors several genes for aminoglycosideases that render it less susceptible to compounds such as gentamicin. Of these AAC(6′)‐Iz, AAC(6′)‐Iak, and APH(3′)‐llc were identified in the sequenced isolates. However, AAC(6′)‐Iak has not been confirmed as conferring gentamicin resistance, while AAC(6′)‐Iz only confers low levels of gentamicin resistance (Lambert et al., 1999; Li, 2003; Tada et al., 2014), and as APH(3′)‐llc was identified in gentamicin susceptible isolates its role in conferring gentamicin resistance is unlikely. HtpX and ClpA aminoglycoside determinants (Huang et al., 2018) were also detected in all sequenced isolates, potentially ruling these out in the conferral of gentamicin resistance. Other mechanisms which may be contributing to the observed gentamicin resistance include multidrug efflux pumps, such as those belonging to the RND family (Venter et al., 2015), and outer membrane changes (Gil‐Gil et al., 2020). More research assessing expression levels of the aforementioned determinants, especially efflux pumps, would be needed to elucidate the mechanisms of gentamicin resistance employed by the isolates evaluated in this study.

Assessment of colistin resistance revealed that 49.3% (*n* = 36/73) of all isolates were found to have MICs of > 4 mg/L. Isolate Sm0581 was found to be highly colistin‐resistant (MIC of 128 mg/L), with others like Sm3119 also displaying high resistance levels with an MIC of 64 mg/L. Reports investigating colistin resistance in *S. maltophilia* have shown that these organisms carry two MCR‐like enzymes, encoded by the *eptA* genes (Li, Liu, et al., 2019; Xie et al., 2021). These phosphoethanolamine transferases are chromosomal and are thought to be able to give rise to colistin resistance genes through various pathways (Gaballa et al., 2022). Evaluation of these revealed that all isolates were found to carry the genes endocing the EptA1 and EptA2 enzymes, except for Sm6012 which was missing EptA1. Genomic analysis of the environmental R551‐3 isolate also revealed a lack of EptA1, with both isolates shown to be susceptible to colistin (Sm6012 MIC 0.5 mg/L, R551‐3 MIC 0.5 mg/L (Youenou et al., 2015)). It is, however, unclear as to the effect these may have on colistin resistance, if any, as isolates carrying both *eptA* genes show variable MICs against colistin, with those determined to be sensitive showing MICs of 4 mg/L, also carrying both enzymes. Analysis of other known determinants which can confer colistin resistance, such as *phoPQ, pmrAB* and *ramA* was also carried out, however, no mutations were observed within these which may have contributed to colistin resistance (data not shown).

Genomic analysis of the mechanisms contributing to trimethoprim‐sulfamethoxazole resistance was undertaken in the resistant Sm2017, Sm5341 and Sm2128 *S. maltophilia* isolates (MICs of ≥ 4 mg/L). In addition to *sul* and *dfrA* genes, which encode enzymes for sulfonamide resistance and dihydrofolate reductase enzymes, respectively, the *qac/smr* genes, encoding small multidrug resistance proteins, were also screened. These determinants have been linked to trimethoprim‐sulfamethoxazole resistance (Neela et al., 2012). However, none of the resistant isolates were positive for *sul* or *dfrA* genes, and although found to be carrying both *smr* and *qacE* genes, these were also identified in susceptible isolates. The number of trimethoprim‐sulfamethoxazole‐resistant isolates (11.0%, *n* = 8/73) is concerning as this compound is currently recommended as the first choice of treatment for *S. maltophilia* infections (Tamma et al., 2022), with increasing rates of resistance complicating and limiting treatment options.

Finally, none of the sequenced *S. maltophilia* isolates were found to harbor plasmids. This finding is in accordance with other studies, which have shown that *S. maltophilia* does not rely on plasmids as a major mechanism for the dissemination and acquisition of AMR genes, as such their presence in these organisms is limited (Brooke, 2012; Chang, 2004).

### 3.4 *S. maltophilia* exemplify a high degree of genetic diversity which continues to evolve

Molecular typing and assessment of *S. maltophilia* population structure has traditionally involved the use of pulsed‐field gel electrophoresis (PFGE) but has now shifted towards multilocus sequence typing (MLST) (Kaiser et al., 2009). The use of different typing methods makes the assessment of the global population structure, clonality and identification of disease‐specific clones challenging. In this study, MLST analysis of the sequenced *S. maltophilia* isolates revealed three novel MLSTs, which included both river strains, Sm0008_R (ST864), Sm0010_R (ST865) and Sm3119 (ST863), isolated from Facility 1. New allele sequences were submitted to the MLST database, hosted by the University of Freiburg, Germany (https://pubmlst.org/bigsdb?db=pubmlst_smaltophilia_isolates). Sm3147 and Sm5341 were found to belong to ST24, whilst the remaining isolates were shown to have varying MLST profiles which included ST41, ST45, ST180, ST183, ST199, ST233 and ST487. According to the PubMLST *S. maltophilia* database (https://pubmlst.org; accessed 2nd February 2023), the aforementioned STs have been isolated from clinical samples of human origin with ST24 and ST233 isolates recovered from blood and all of the ST identified in this study isolated from sputum samples. Isolation from sputum samples is not surprising as *S. maltophilia* ST183 isolates found in cystic fibrosis individuals suffering from chronic pulmonary infections (Esposito et al., 2017). Another ST previously identified in relation to cystic fibrosis is ST199. Both ST183 (Facility 1) and ST199 (Retirement) *S. maltophilia* isolates recovered in this study were found to be MDR.

**Figure 5:**
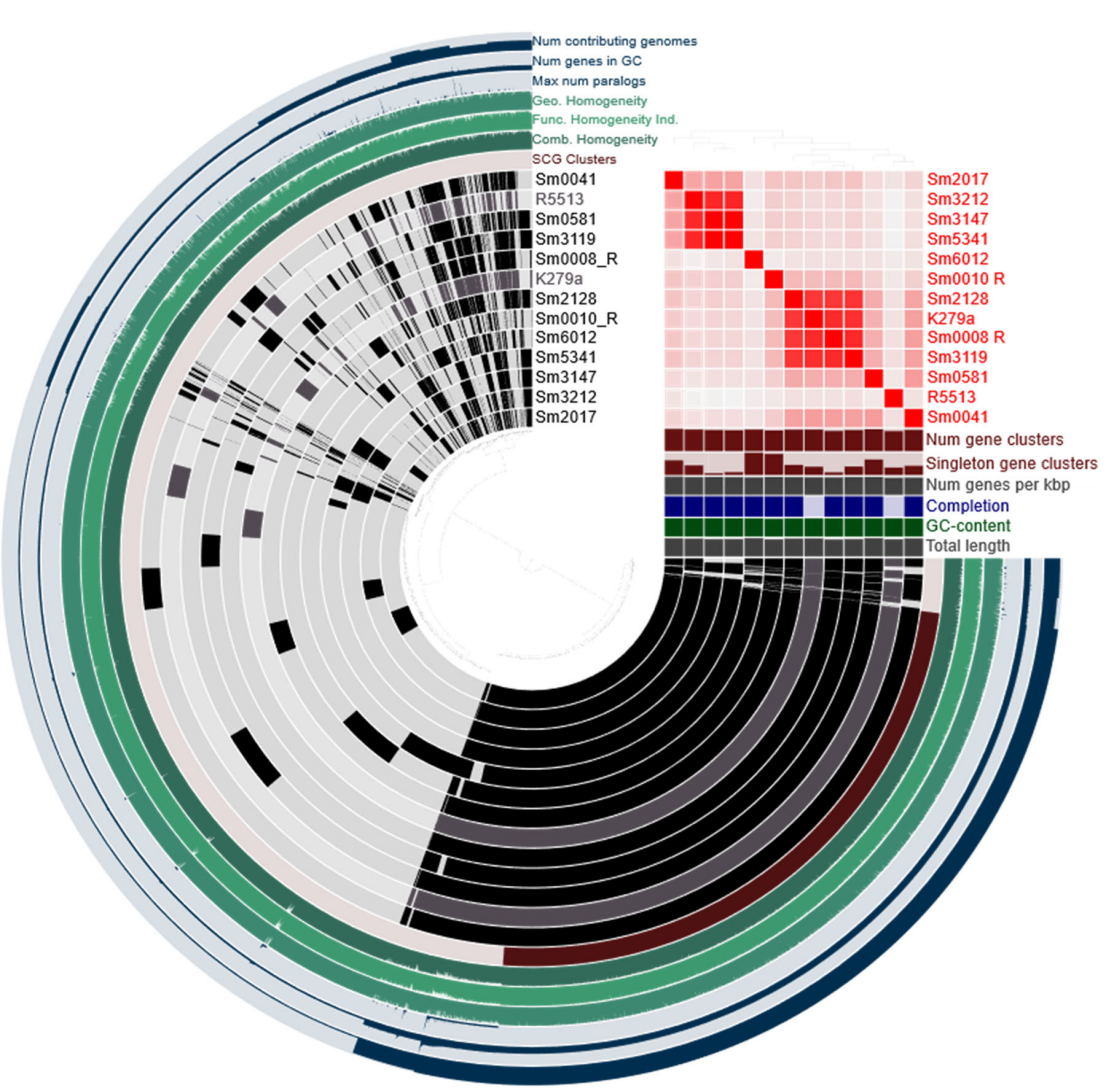
The pangenomes of sequenced *Stenotrophomonas maltophilia* isolates illustrate diversity as demonstrated by their accessory genomes (reference genomes [shaded in gray] include: *S. maltophilia* K279a [accession No. AM743169], and R551‐3 [accession No. NC_011071]). An ANI percent identity heatmap (red squares indicate higher similarity between genomes), is provided with the number of gene clusters and singleton gene clusters bar charts included below. Additional statistics related to this analysis include the number of genes per kbp (gray bar chart), completion (blue bar chart), GC content (green bar chart) and total length (bottom gray bar chart) are also provided. Finally, the outer 6 layers correspond to the number of contributing genomes, number of genes per GC, maximum number of paralogs, and genomic, functional and combined homogeneity indexes.

A pangenome analysis of the sequenced *S. maltophilia* isolates and the two reference genomes (K279a and R551‐3) revealed 1873 core genes (found in > 99% genomes), 4807 shell genes (found in 15%–95%) and 9256 cloud genes (found in less than 15% of genomes), revealing isolates carrying highly individual gene sets. Average nucleotide identity (ANI) values determined for these isolates (Table S1) indicate highly similar lineages for Sm008_R, Sm3119 (Facility 1) and Sm2128 (Retirement) (ANI values > 95%), and Sm3212 (Retirement), Sm3147 (Facility 1) and Sm5341 (Facility 3), all which were isolated from different environments (Figures 4 and 5). Of particular interest are isolates Sm3147 and Sm5341, which despite being isolated from two different environments (wastewater and sink) and facilities (Figure 4) show an ANI of 100%, suggesting a common origin.

The lowest ANIs, indicative of a distinct lineage, were observed for Sm6012 (Facility 2) and were found to be comparable to the low ANIs obtained for the environmental reference strain R551‐3. The Sm6012 *S. maltophilia* isolate was also found to carry the greatest number of singleton gene clusters (gene clusters which are only present in a single genome), which represent rare chromosomal genetic variations and provide a measure of geographic genetic diversity (Cubry et al., 2017). In this study, both Sm6012 and Sm0010_R were observed to display high numbers of singleton gene clusters, with other isolates all displaying single‐copy core genes indicating the high genetic diversity which is characteristic of this species (Gröschel et al., 2020).

**Figure 6:**
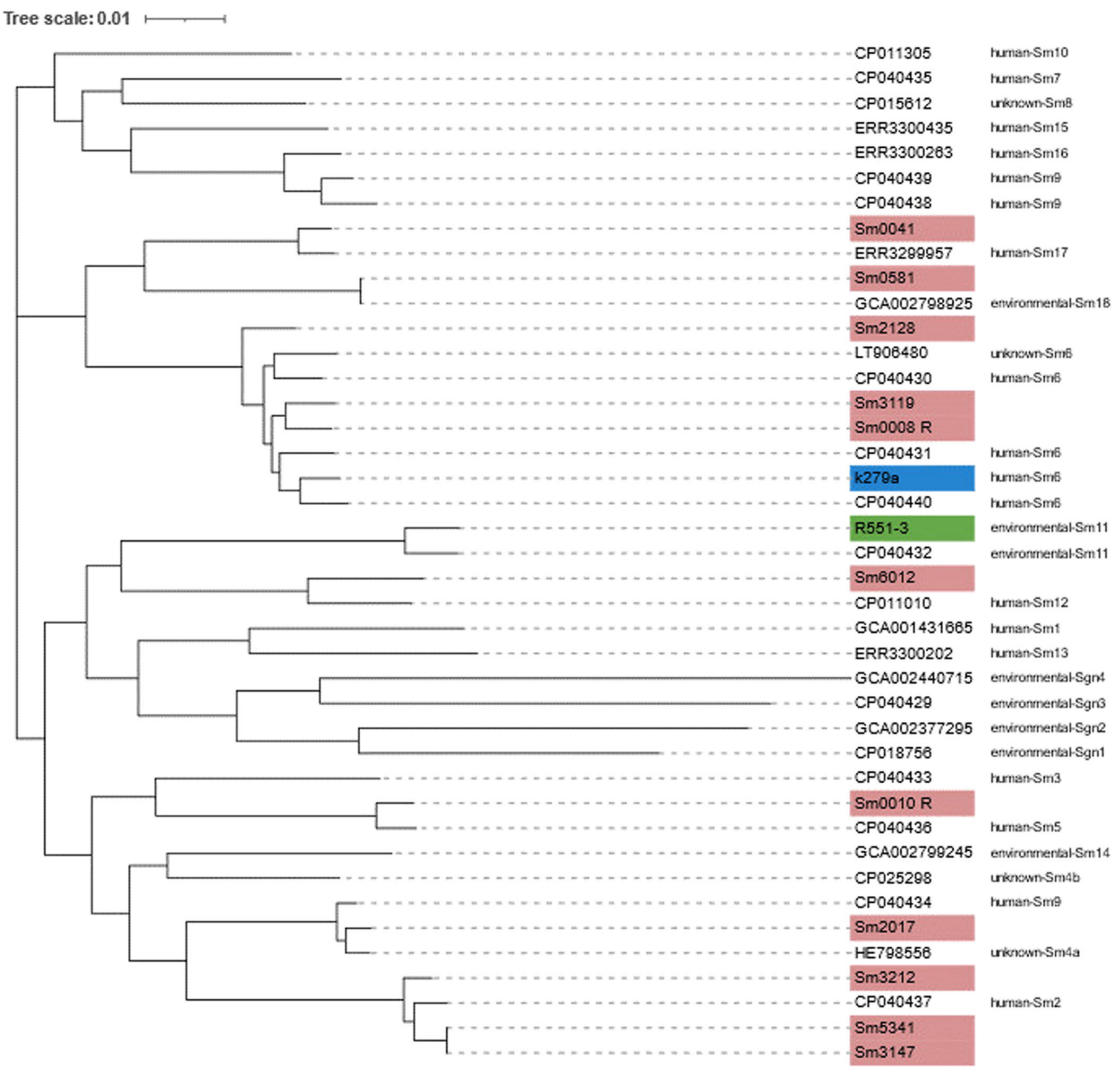
A core genome‐based maximum likelihood phylogenetic tree (*n* = 41) illustrating clustering of sequenced *Stenotrophomonas maltophilia* isolates from this study (pink), two reference strains (K279a (blue) and R551‐3 (green) with isolates representing each of the 23 lineages as determined by Gröschel et al. (2020).

Recent studies aimed at inferring the global population structure of *S. maltophilia* identified 23 monophyletic lineages, including the Sm6 lineage, which is comprised of the most frequently isolated human strains linked with resistance and virulence genes (Gröschel et al., 2020). To ascertain the lineages associated with the *S. maltophilia* assessed in this study, a phylogenetic tree (core genomes) and ANI values were determined for these using a representative strain of each of the 23 lineages (Figure 6). The MDR isolates Sm0008‐R and Sm3119 were clustered with isolates belonging to the Sm6 lineage, along with the clinical K279a strain. Sm3212, Sm3147 and Sm5341, were clustered with the Sm2 lineage, which was also associated with isolates recovered from human samples. Two isolates, Sm0041 and Sm0581 were clustered with Sm17 and Sm18 lineages, respectively, which according to the 2020 study have not previously been identified in Australia, possibly indicating the continual spread of *S. maltophilia*. Finally, Sm2017 clustered with Sm4a, a lineage of isolates identified to be missing *bla*_L1_, a gene encoding the L1 metallo‐beta‐lactamase. However, Sm2017 was found to carry both L1 and L2 beta‐lactamases. Upon further assessment, isolate GCA_000284595.1 (accession no. HE798556.1), assigned to the Sm4a linage in the original study carried out by Gröschel et al. (2020), and used here to represent this lineage, was reviewed and its sequence found to also be carrying the *bla*_L1_ gene (locus tag: SMD_2343).

## 4 DISCUSSION

*S. maltophilia* is a ubiquitous, emerging, opportunistic pathogen which is increasing in frequency of isolation in clinical settings and is associated with significant mortality rates in immunocompromised patients (Flores‐Treviño et al., 2014; Tan et al., 2008). Treatment options are limited due to their high intrinsic resistance against many currently available antimicrobial agents, including carbapenems and other beta‐lactams (Looney et al., 2009). As an emerging opportunistic pathogen, much is still unknown about *S. maltophilia's* mechanisms of resistance. Among the well‐characterized mechanisms are those for beta‐lactam resistance (Crowder et al., 1998; Walsh et al., 1997), however, as can be seen in this study, more research needs to be carried out to elucidate mechanisms of resistance to such agents as colistin. There also appears to be a dearth of information pertaining to biocide tolerance with few studies assessing biocide mechanisms of tolerance within *S. maltophilia* (Gil‐Gil et al., 2020).

The assessment of 73 *S. maltophilia* isolated from three residential aged care facilities and a Retirement village located in Adelaide, South Australia, revealed high levels of beta‐lactam resistance in all of the isolates assessed here. A comparison between sites of isolation revealed that Facility 1 isolates have higher levels of resistance against colistin and gentamicin, while Facility 3 isolates displayed higher trimethoprim‐sulfamethoxazole resistance. It is difficult to ascertain the factors contributing to different resistance patterns found between facilities, as all three facilities and the Retirement village are run by the same care provider. Notable differences between facilities include resident numbers, with Facility 1 catering for a larger number of residents (170 beds, compared to both Facility 2 (70 beds) and Facility 3 (58 beds). However, even though Facility 3 caters for the smallest number of residents the *S. maltophilia* isolates recovered from this facility did not only display higher trimethoprim‐sulfamethoxazole resistance but were also found to be generally more resistant to cefepime and gentamicin when compared with Facility 2 isolates. Although it is difficult to extrapolate specific factors contributing to resistance within each facility, the development of resistance may be influenced by the environment itself. Within RACFs antibiotics and biocides are used in large quantities (Stuart et al., 2012; Sundvall et al., 2015), potentially selecting for increased resistance in *S. maltophilia* (Gil‐Gil et al., 2020).

Evaluation of the susceptibility profiles determined in this study revealed high cephalosporin and carbapenem resistance, which has been well‐documented in other studies (Chang et al., 2015; Crossman et al., 2008; Gould et al., 2006). Gentamicin resistance was also observed, with the presence of known aminoglycosidases such as AAC(6′)‐lak and AAC(6′)‐lz identified in gentamicin‐resistant isolates. However, high gentamicin resistance was observed in *S. maltophilia* Sm0010_R (MIC of 64 mg/L), which did not carry the genes encoding these enzymes, suggesting an alternative gentamicin resistance mechanism, such as the employment of drug efflux pumps. MDR efflux pumps play a major role in AMR in *S. maltophilia*, with RND pumps identified as playing a key role in both intrinsic and acquired resistance (Alonso & Martínez, 2000; Chang et al., 2015; Li, Zhang, et al., 2019; Sánchez & Martínez, 2018). Eight RND efflux pumps have been identified thus far, with SmeABC and SmeDEF found to play a role in intrinsic resistance (Alonso & Martínez, 2000; Li et al., 2002; Zhang et al., 2001). Overexpression of these can also lead to acquired resistance, with SmeDEF overexpression leading to trimethoprim‐sulfamethoxazole resistance (Alonso, 2004; Sánchez & Martínez, 2018), and SmeYZ overexpression to gentamicin resistance (Calvopiña et al., 2020). Finally, the acquisition of mutations within such RND efflux pumps as SmeH, a component of the SmeGH efflux system, has been shown to lead to elevated ceftazidime resistance (Blanco et al., 2019), however, these were not observed in ceftazidime‐resistant isolates assessed in this study.

Although efflux pumps have been shown to play a role in colistin resistance in Gram‐negative bacteria (Lin et al., 2017; Puja et al., 2020), current evidence of their involvement in *S. maltophilia* is based on a study that used the protonophore carbonyl cyanide 3‐chlorophenylhydrazone (CCCP). This study revealed that upon the addition of CCCP, decreased colistin resistance was observed in *S. maltophilia*, as well as in other Gram‐negative bacteria, eluding to the involvement of efflux pumps in colistin resistance (Ni et al., 2016). However, no actual efflux pump/s have been identified thus far contributing to colistin resistance. A recent study has also identified two putative colistin‐resistance determinants shown to have high homology with the mobile colistin‐resistant determinant, *mcr‐1*, in *Stenotrophomonas* sp. strain G4 isolated from wastewater (Li, Liu, et al., 2019). These MCR‐like determinants show high homology with the phosphoethanolamine transferases EptAs, which are also being evaluated in their role in colistin resistance. Recently an intrinsic phosphoethanolamine transferase, EptA_AM was found to contribute to colistin resistance in an *Acinetobacter modestus* isolated in Japan, with transformation of *Escherichia coli*, *Klebsiella pneumoniae* and *Enterobacter cloacae* with the *eptA_AM* gene and its promoter leading to elevated colistin resistance in these isolates (Sakuma et al., 2023). Increasing colistin resistance rates within this organism, reported to be 8% in 1996% and 45% in 2013 in Argentina (Rodríguez et al., 2014) are a concern, as colistin is a last resort antibiotic reserved for the treatment of carbapenem‐resistant infections.

Once again illustrating a significant knowledge gap, little is also known regarding biocide tolerance within *S. maltophilia*. With no ECOFF values determined for biocides, it is difficult to assess biocide tolerance (obtaining a MIC value above the ECOFF) in *S. maltophilia*. However, using the ECOFF values determined in this study, biocide tolerance appears to be low amongst the isolates assessed here, with only one isolate shown to tolerate benzalkonium chloride (MIC 128 mg/L) and one triclosan (MIC 256 mg/L). These results mirror previous studies which show that biocide resistance appears to be uncommon in natural populations of clinically relevant microorganisms such as *E. coli* and *K. pneumoniae*, amongst others (Morrissey et al., 2014). Nonetheless, the high ECOFF values determined here illustrate the ability of *S. maltophilia* to generally tolerate high levels of triclosan, commonly observed in *P. aeruginosa* (Zhu et al., 2010). Studies in *S. maltophilia* revealed that subinhibitory concentrations of triclosan can induce transient overexpression of the SmeDEF efflux pump (Sánchez et al., 2015). As triclosan is commonly used in antiseptics and disinfectants, its use within healthcare settings such as RACFs is potentially high and may select for triclosan‐tolerant *S. maltophilia*. Overexpression of SmeDEF may also lead to reduced susceptibility of *S. maltophilia* against antibiotics such as chloramphenicol and certain quinolones, in addition to other antibiotics which are substrates of this drug efflux pump (Alonso & Martínez, 2000; García‐León et al., 2014). As such, more research is needed to evaluate biocide tolerance in *S. maltophilia* and to ascertain their biocide tolerance mechanisms.

As an emerging opportunistic pathogen, genome‐based population studies have been conducted to assess transmission, clonality and global spread of *S. maltophilia* (Gröschel et al., 2020; Patil et al., 2018; Vinuesa et al., 2018). Such research is complicated as not only is *S. maltophilia* genetically diverse, leading to the formation of a so‐called *S. maltophilia* complex (Ochoa‐Sánchez & Vinuesa, 2017) but multiple taxonomic revisions have hindered classification, potential assessment of distribution, and assessment of possible associations between species and their habitat, resistance phenotypes and pathogenicity potential (Adamek et al., 2011). In this study, using the recently established 23 monophyletic lineages (Gröschel et al., 2020), isolates clustering with Sm17 and Sm18 lineages may be indicative of the continual global spread of *S. maltophilia*, with isolates belonging to these lineages not previously detected in Australia. The clustering of isolates assessed in this study with both the Sm6 and Sm2 lineages, which represent isolates associated with human/clinical samples that are linked to virulence and resistance, is concerning given their isolation from RACFs which house a vulnerable population.

A limitation of this study and others purporting *S. maltophilia* resistance is the lack of guidelines established regarding susceptibility methods and the lack of ECOFF and clinical breakpoints available for this organism for many commonly used antibiotics (Chang et al., 2015; Hombach et al., 2012; Juhász et al., 2014). According to EUCAST guidelines, reasons for the absence of established antimicrobial susceptibility methods and subsequent determination of breakpoints are based on the tendency of results to be affected by incubation temperature, use of specific culture medium and techniques such as disk diffusion, broth dilution, gradient MIC tests, amongst others (EUCAST, 2012). The lack of guideless makes the comparison of global surveillance studies and *S. maltophilia* susceptibility patterns difficult and can lead to complications when reviewing clinical data (Chang et al., 2015).

Nonetheless, as demonstrated in this study, the need to further our understanding of *S. maltophilia* is becoming urgent, as the incidence of MDR isolates is growing worldwide (Gröschel et al., 2020; Kumar et al., 2020). The presence of this MDR opportunistic pathogen in RACFs is worrisome, with isolates which are not only beta‐lactam resistant but also gentamicin, colistin and trimethoprim‐sulfamethoxazole resistant identified in this study. Considering that most residents are immunocompromised and frequently exposed to antibiotics, RACFs represent an environment that can facilitate the continual development of bacterial resistance. With its high genetic diversity and MDR phenotype, *S. maltophilia* can adapt and prosper in such an environment. As such, the presence of *S. maltophilia* in RACFs signifies a potential problem, with current limited treatment options available, MDR *S. maltophilia* may leave us with no therapeutic choices available to treat infections caused by this opportunistic pathogen.

## 5 CONCLUSION

In this study we assessed *S. maltophilia* isolates recovered from residential aged care facilities (RACFs), where antibiotic overuse has been reported, thus creating a selective environment for the development of bacterial resistance. *S. maltophilia* analyzed here were found to be multidrug‐resistant, with colistin non‐wild type (49.3%, 36/73), and trimethoprim‐sulfamethoxazole resistant (11%, 8/73) isolates identified in this study. The presence of these in RACFs is concerning as *S. maltophilia* continues to adapt and emerge with isolates clustering with known lineages Sm17 and Sm18, not previously identified in Australia, observed here, possibly indicating the continual spread of these organisms.

## AUTHOR CONTRIBUTIONS

**Sylvia A. Sapula**: Conceptualization; methodology; investigation; validation; formal analysis; data curation; visualization; writing—original draft; writing—review and editing; supervision; project administration. **Bradley J. Hart**: Methodology; investigation; validation; formal analysis; visualization; writing—review and editing; writing—original draft; data curation. **Naomi L. Siderius**: Writing—review and editing; investigation. **Anteneh Amsalu**: Writing—review and editing; investigation. **Jack M. Blaikie**: Writing—review and editing; investigation. **Henrietta Venter**: Conceptualization; formal analysis; writing—review and editing; project administration; resources; supervision; funding acquisition.

## CONFLICT OF INTEREST STATEMENT

None declared.

## ETHICS STATEMENT

None required.

## Supporting information

Supporting information.

## Data Availability

Draft whole genome sequences of the 11 *S. maltophilia* isolates sequenced in this study are available in the NCBI database under BioProject ID: PRJNA907874, with the following accession numbers; Sm3119: JAPTFY000000000, Sm6012: JAPTFX000000000, Sm3147: JAPTFW000000000, Sm2017: JAPTFV000000000, Sm3212: JAPTFU000000000, Sm0581: JAPTFT000000000, Sm5341: JAPTFS000000000, Sm0041: JAPTFR000000000, Sm2128: JAPTFQ000000000, Sm0008_R: JAPTFP000000000, Sm0010_R: JAPTFO000000000: https://www.ncbi.nlm.nih.gov/bioproject/PRJNA907874
